# Automatic Detection of Obstructive Sleep Apnea Events Using a Deep CNN-LSTM Model

**DOI:** 10.1155/2021/5594733

**Published:** 2021-03-22

**Authors:** Junming Zhang, Zhen Tang, Jinfeng Gao, Li Lin, Zhiliang Liu, Haitao Wu, Fang Liu, Ruxian Yao

**Affiliations:** ^1^College of Information Engineering, Huanghuai University, Zhumadian, Henan 463000, China; ^2^Henan Key Laboratory of Smart Lighting, Zhumadian, Henan 463000, China; ^3^Henan Joint International Research Laboratory of Behavior Optimization Control for Smart Robots, Zhumadian, Henan 463000, China; ^4^Zhumadian Artificial Intelligence & Medical Engineering Technical Research Centre, Zhumadian, Henan 463000, China; ^5^Academy of Industry Innovation and Development, Huanghuai University, Zhumadian, Henan 463000, China

## Abstract

Obstructive sleep apnea (OSA) is a common sleep-related respiratory disorder. Around the world, more and more people are suffering from OSA. Because of the limitation of monitor equipment, many people with OSA remain undetected. Therefore, we propose a sleep-monitoring model based on single-channel electrocardiogram using a convolutional neural network (CNN), which can be used in portable OSA monitor devices. To learn different scale features, the first convolution layer comprises three types of filters. The long short-term memory (LSTM) is used to learn the long-term dependencies such as the OSA transition rules. The softmax function is connected to the final fully connected layer to obtain the final decision. To detect a complete OSA event, the raw ECG signals are segmented by a 10 s overlapping sliding window. The proposed model is trained with the segmented raw signals and is subsequently tested to evaluate its event detection performance. According to experiment analysis, the proposed model exhibits Cohen's kappa coefficient of 0.92, a sensitivity of 96.1%, a specificity of 96.2%, and an accuracy of 96.1% with respect to the Apnea-ECG dataset. The proposed model is significantly higher than the results from the baseline method. The results prove that our approach could be a useful tool for detecting OSA on the basis of a single-lead ECG.

## 1. Introduction

Obstructive sleep apnea (OSA) is a major sleep-disordered breathing (SDB) syndrome that is an independent risk factor of coronary heart disease, hypertension, and arrhythmia [1]. According to the manual of the American Academy of Sleep Medicine (AASM) [2], OSA in adults is scored when there is a 90% or more reduction in the baseline of the oral and nasal respiration amplitude for 10 s or more, occuring during sleep. This condition is associated with repetitive airflow limitation and sleep fragmentation, decreasing the sleep time and degrading the sleep quality of the OSA patients [3]. OSA not only causes excessive daytime neurocognitive deficits, drowsiness, depression, fatigue, and heart stroke [4–6] but can also cause a brain stroke, high blood pressure, arrhythmias, myocardial infarction, and ischemia [7–9]. According to the AASM [2], polysomnography (PSG) is considered to be the gold standard for OSA detection, which is based on a comprehensive evaluation of the sleep signals [10]. PSG involves overnight recording of the patient and the measurement of many signals using the sensors attached to the body, e.g., an electroencephalogram (EEG), electromyogram (EMG), electrocardiogram (ECG), and electrooculogram (EOG), to monitor the respiratory effort and other biophysiological signals [1]. After collecting the PSG data, physicians inspect them using statistical tools to score the OSA events.

However, PSG has several disadvantages. First, patients need to sleep in the hospital for at least one night, which consumes a considerable amount of time and is expensive. Furthermore, many patients cannot sleep well in hospitals. Second, many electrodes have to be connected to the body of a patient. These electrodes will interrupt their sleep, which will result in the deviation of the measurement results. Therefore, it is important to develop methods that can reliably diagnose OSA with a few signals and that can be used at home. According to Mietus and Peng [11], the heart beat interval of patients fluctuates periodically during the occurrence and recovery of OSA. Zarei and Asl [12] indicated that significant changes in heart rate or abnormal activities of the heart may indicate OSA. Additionally, according to our clinical research, patients' compliance is very low when they wear the pressure transducer sensor to obtain the oral and nasal respiration. Patients often pull out the nasal cannula. Therefore, when compared with the ECG signal, nasal airflow data can be unstable due to lead falling off. Hence, in this study, we use ECG signals to detect OSA events.

Traditional visual OSA scoring is a very tedious and time-consuming process for a physician to conduct. Therefore, many alternative OSA detection methods have been developed [13]. These methods were based on biosignals such as the respiratory [14], snoring [15–17], SpO2 [8, 9, 18], and ECG [12, 19–24] signals, and many authors have obtained a high performance level in terms of OSA detection. However, almost all these methods involved data preprocessing, feature extraction, feature selection, and classification. Although feature extraction is essential to ensure good performance, this process requires considerable domain expertise and is particularly limited to high-dimensional data [25].

Deep learning is an attractive alternative because it can automatically learn and extract features from raw data and can be merged with a classification procedure. In particular, convolutional neural networks (CNNs), which are a popular deep-learning model, have gained considerable success owing to their excellent performance in various domains, including visual imagery [26], speech recognition [27], and text recognition [28]. CNNs have also been applied to biosignal classification problems. For example, in our previous study [29], a CNN can be used to score the sleep stages. Banluesombatkul et al. [30] used metalearning classify sleep stages. Piriyajitakonkij et al. [31] proposed a SleepPoseNet to recognize sleep postures. An event-related potential encoder network was applied to ERP-related tasks [32]. Wilaiprasitporn et al. [33] used a deep-learning approach to improve the performance of affective EEG-based person identification. Recently, some models based on CNNs have been employed to detect OSA. Urtnasan et al. [25] proposed a method for the automated detection of OSA from a single-lead ECG using a CNN. Ho et al. [10] developed an approach for OSA event detection using a CNN and a single-channel nasal pressure signal. Banluesombatkul et al. [34] used a CNN to extract ECG signal features and fully connected neural networks for OSA events detection. McCloskey et al. [35] used a CNN and wavelets to analyze the nasal airflow and detect the OSA events. However, most of these methods score OSA events by minute-by-minute analysis. According to the AASM ruler [2], OSA events occur in 10 s or more. Therefore, minute-by-minute analysis will lose some OSA events. At the same time, the duration of each OSA event is different. Multiple OSA events can occur as briefly within only a single minute (i.e., one epoch); at times, one OSA event can be prolonged over multiple epochs. Therefore, it is difficult to detect complete OSA events for these methods.

According to Guilleminault et al. [36], there is a relation between the OSA events and heart rate variability. They indicated that the heart rate decelerates at the beginning of an OSA event and that it suddenly increases when normal breathing is resumed [36]. Because long short-term memory (LSTM) maintains internal memory and utilizes feedback connections to learn temporal information from sequences of inputs, in this study, we propose a new method for OSA detection using the CNN and LSTM. The LSTM [37] is used to learn these dependencies, such as the transition rules employed by physicians, to identify future OSA events from previous ECG epochs. To detect complete OSA events, a window overlapping method is required to accurately detect the OSA events, which can identify the start and end positions of the event. Therefore, the proposed method can alert for OSA events of long duration, which will reduce the rate of sudden death caused by OSA events [38].

This study is organized as follows: the datasets are presented in Section 2, and the methods are presented in Section 3. The experimental results and discussion are presented in Section 4, and Section 5 concludes this study.

## 2. Dataset and Preprocessing

The Apnea-ECG dataset [39], downloaded from https://www.physionet.org/content/apnea-ecg/1.0.0, was used to evaluate the proposed approach. The dataset comprises 70 PSG recordings, among which 35 are used in the training set and 35 are used in the test set. The training set was used to update the parameters of the proposed model, and the test set was used to perform independent performance assessments. Each recording contains a continuous digitized ECG signal, a set of apnea annotations (derived by human experts on the basis of the simultaneously recorded respiration and related signals), and a set of machine-generated QRS annotations. The sampling rate for the ECG was 100 Hz with a 12 bit resolution. The records contain variable lengths from 7 to 10 hours. The age of the subjects is between 27 and 63 years, and their weights are 35–135 kg.

First, according to Urtnasan et al. [25], a Chebyshev type-II band-pass filter (5–11 Hz) was used to remove undesirable noise from the single-lead ECG data. Second, the data were segmented into epochs (10 s long) to train the proposed model.  Table 1 presents the distribution of all the epochs in the training and test sets. Abnormal epoch means an OSA event.

## 3. Methods

### 3.1. Convolutional Neural Network

In this study, we used a one-dimensional (1D) CNN to classify the ECG signals. The CNN comprised convolutional, pooling, and fully connected layers. The net input of neuron *j* in layer *l* is defined as follows:

(1) $$\begin{array}{c} Z_{j}^{l}=\sum_{i\in M_{j}}w_{j,i}^{l}*x_{i}^{l-1}+b_{j}^{l}, \end{array}$$

where *M*_*j*_ represents the selection of input maps, *w*_*j*,*i*_ denotes the weight or the filter associated with the connection between neurons *j* and *i*, *x*_*i*_^*l*−1^ is the output signal from neuron *i* in layer *l* − 1, *b*_*j*_^*l*^ is the bias associated with neuron *j* in layer *l*, and (^∗^) denotes vector convolution. To acquire an output map, an activation function is required as follows:

(2) $$\begin{array}{c} x_{j}^{l}=f\left(z_{j}^{l}\right). \end{array}$$

When compared with other activation functions, a rectified linear unit (ReLU) exhibits robust training performance. Hence, in this study, we used ReLU as the activation function for the output maps, which can be expressed as follows:

(3) $$\begin{array}{c} f\left(z_{j}^{l}\right)=\max\left(0,z_{j}^{l}\right). \end{array}$$

After the convolutional layer, a pooling layer was placed, which was used to reduce the dimensions of the feature maps, network parameters, and the computational cost associated with successive layers using specific functions to summarize the subregions, such as by considering the average value or the maximum value. Additionally, the pooling layer allowed the CNN to learn features that were scale invariant or can be attributed to the orientation changes [40]. The pooling operation consisted of sliding a window across the previous feature map. Herein, max pooling was used after the convolutional layer was activated. Finally, a dense layer, which was generally used in the final stages of the CNN, was fully connected to the outputs of the previous layers.

### 3.2. Batch Normalization

During the training of a CNN, a change in the distribution of the inputs of each layer will affect the outputs of all the succeeding layers. This can result in difficulty when attempting to train models with saturated nonlinearities [41]. Therefore, batch normalization (BN) was used to solve this problem.

Suppose *X* = {*x*_1_,  *x*_2_, ⋯, *x*_*d*_} is the input to a layer with dimension *d*. The corresponding minibatch is *mb*. The mean of all the inputs in the same minibatch can be expressed as follows:

(4) $$\begin{array}{c} \mu =\frac{1}{mb}\sum_{i=1}^{mb}x_{i}. \end{array}$$

The variance of the input in a minibatch can be expressed as follows:

(5) $$\begin{array}{c} \sigma^{2}=\frac{1}{mb}\sum_{i=1}^{mb}{\left(x_{i}-\mu \right)}^{2}. \end{array}$$

Therefore, BN can be expressed as follows:

(6) $$\begin{array}{c} y_{i}=\gamma x_{i}^{∼}+\beta , \end{array}$$

where $x_{i}^{∼}=x_{i}-\mu /\sqrt{\varepsilon +\sigma^{2}}$, *γ*, and *β* are learnable parameters. The training efficiency of a CNN can be improved using BN. At the same time, BN helps the CNN to train faster and provides high accuracy [41].

### 3.3. Long Short-Term Memory

LSTM controls the cell state via three gates, i.e., a forgetting gate, an input gate, and an output gate. The output features obtained from the previous dense layer of a CNN layer are passed to the gate units. The memory cells constituting the LSTM update their states via the activation of each gate unit controlled to a continuous value between 0 and 1. The hidden state of the LSTM cell *h*_*t*_ is updated after every *t* steps. The input gate, forget gate, and output gate can be written as shown in equations (7)–(9) [37], respectively.

(7) $$\begin{array}{c} i^{t}=\text{sigmoid}\left(W_{ni}X^{t}+W_{hi}X^{t-1}+W_{ci}{}^{\circ}c^{t-1}+b_{i}\right), \end{array}$$

(8) $$\begin{array}{c} f^{t}=\text{sigmoid}\left(W_{nf}X^{t}+W_{hf}h^{t-1}+W_{cf}{}^{\circ}c^{t-1}+b_{bf}\right), \end{array}$$

(9) $$\begin{array}{c} o^{t}=\text{sigmoid}\left(W_{no}X^{t}+W_{ho}h^{t-1}+W_{co}{}^{\circ}c^{t}+b_{bo}\right), \end{array}$$

where ° represents point-wise multiplication.

The cell states and hidden states can be expressed using equations (10) and (11), respectively.

(10) $$\begin{array}{c} c^{t}=f^{t}\circ c^{t-1}+i^{t}\circ \text{sigmoid}\left(W_{nc}X^{t}+W_{hc}h^{t-1}+b_{bc}\right), \end{array}$$

(11) $$\begin{array}{c} h^{t}=o^{t}\circ \text{sigmoid}\left(c^{t}\right). \end{array}$$

The CNN and LSTM can be used as backpropagation algorithms to update the parameters of the model during training.

## 4. Experiments

### 4.1. Statistical Evaluation Methods

In this study, we use the kappa coefficient (KP) [42], which is a robust statistical measure of the inter-rater agreement, to evaluate the performance of our method. Additionally, the total accuracy (TAC), sensitivity (SE), specificity (SP), positive predictive value (PPV), and negative predictive value (NPV) were calculated according to an epoch-by-epoch analysis as follows:

(12) $$\begin{array}{c} \text{TAC}=\frac{\text{TP}+\text{TN}}{\text{TP}+\text{FN}+\text{FP}+\text{TN}}\%, \end{array}$$

(13) $$\begin{array}{c} \text{Sensitivity}=\frac{\text{TP}}{\text{TP}+\text{FN}}\%, \end{array}$$

(14) $$\begin{array}{c} \text{Specificity}=\frac{\text{TN}}{\text{TN}+\text{FP}}\%, \end{array}$$

(15) $$\begin{array}{c} \text{PPV}=\frac{\text{TP}}{\text{FP}+\text{TP}}\%, \end{array}$$

(16) $$\begin{array}{c} \text{NPV}=\frac{\text{TN}}{\text{TN}+\text{FN}}\%, \end{array}$$

where TP, TN, FP, and FN denote the true positive, true negative, false positive, and false negative, respectively. We implement our experiments on a workstation with a GeForce GTX2060 GPU in a Windows environment. The TensorFlow framework is used to train the proposed model.

### 4.2. The Proposed Deep Model Architecture and Parameters

**Figure 1:**
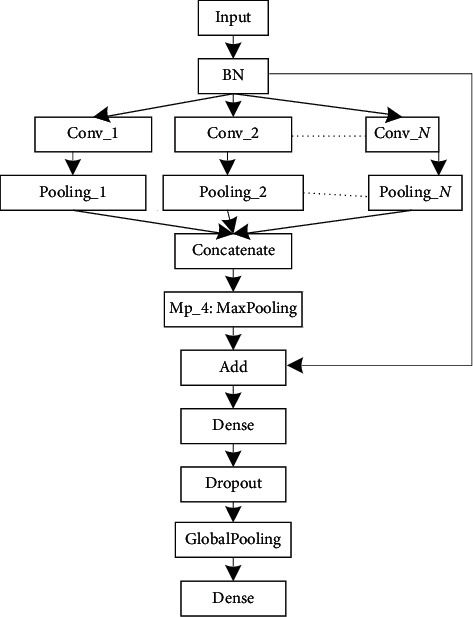
Schematic of the proposed CNN model for the automated detection of OSA.

**Figure 2:**
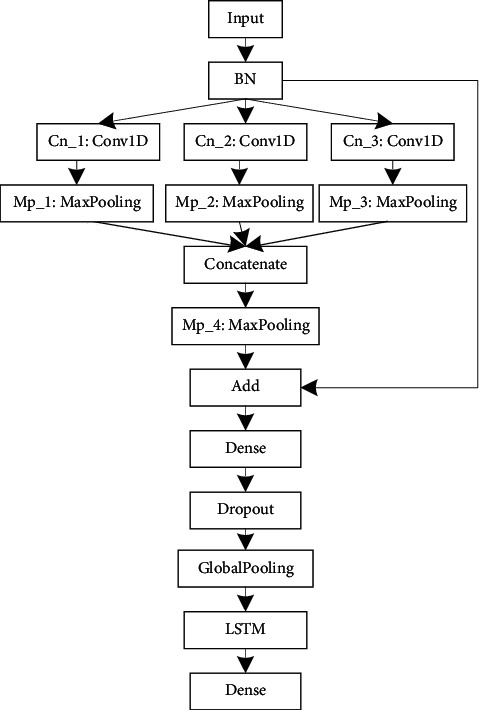
Architecture of the proposed model.

To build an optimal OSA detection architecture, we need to understand the characteristics of the input data. The sampling rate of the ECG was 100 Hz, and the 10 s input dimension was 1000. To extract different scale features, we need to set up different size filters. Therefore, experiments are implemented while varying the filters size of the convolution layer to identify the optimal parameters for automated OSA detection. According to existing study [25, 29], we design a network model, which contains a convolution, BN, pooling, dropout, and dense layer, as shown in Figure 1. *N* denotes the number of the filters. The parameters and results are shown in Table 2. From Table 2, we can see that model_2 performs best and model_1 is the second. However, the parameters of model_2 are large than those of model_1. For portable OSA devices or real-time OSA analysis systems, model_1 is more appropriate. Therefore, model_1 is used to learning the features representation of ECG. To learn the transition rules of OSA, LSTM is used. The proposed model contains the BN, convolutional, pooling, LSTM, and dense layer, as shown in Figure 2.

**Figure 3:**
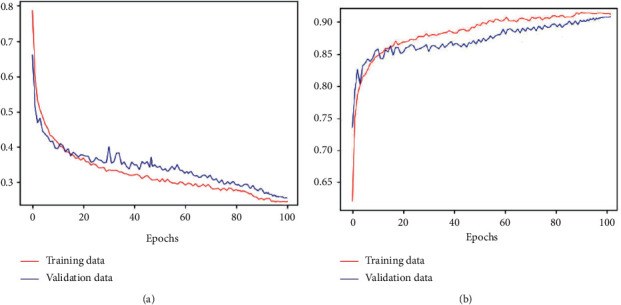
Accuracy and loss of the proposed model for automated OSA detection. (a) Loss curve. (b) Accuracy curve.

**Figure 4:**
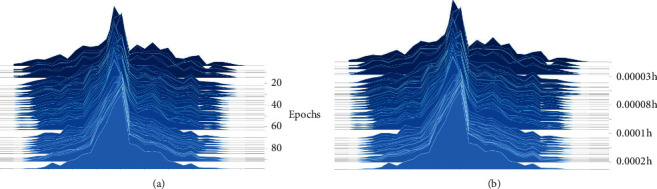
Filters morphology and training time with each epoch. (a) Filter morphology. (b) Training time.

The detailed parameters of the proposed model are presented in Table 3. This table shows the number of filters, the size, and stride in each convolution layer, the size and stride of the kernel in each pooling layer, and the output size of each layer, including the LSTM layer. The batch size is 30, the training epoch is 100, and the learning rate is 0.1.  Figure 3 shows the learning results in terms of accuracy and loss obtained as the number of epochs is varied. The results show that the accuracy and loss reach stable values after several iterations of learning when applied to the validation dataset.  Figure 4 shows the filter morphology and training time with each training epoch. From Figure 4(a), we can see that, after 90 training epochs, the morphology of the filter almost does not change.  Figure 4(b) indicates that the speed of model training is fast.

### 4.3. Performance Results

Table 4 presents the performances of the proposed model for the automated detection of OSA from a single-lead ECG signal. When applied to the test dataset, we obtained a KP of 0.92, an SE of 96.1%, an SP of 96.2%, a TAC of 96.1%, a PPV of 97.6%, and an NPV of 93.8%. As can be seen, the proposed model performed very well for the detection of OSA.

**Figure 5:**
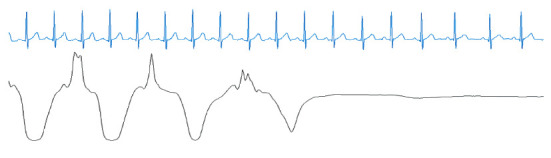
A transition epoch from an NE to an AE. Blue denotes the ECG signal, and black denotes the nasal airflow signal.

**Figure 6:**
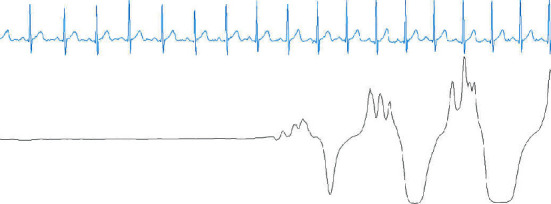
A transition epoch from an AE to an NE. Blue denotes the ECG signal, and black denotes the nasal airflow signal.

**Figure 7:**
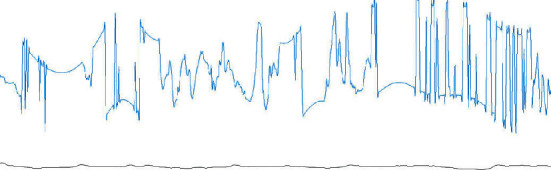
An ECG artifact epoch. Blue denotes the ECG signal, and black denotes the nasal airflow signal.

From Table 4, we can observe that 3.9% of the AEs were misclassified as NEs and that 3.8% of the NEs were misclassified as AEs. According to our research, these misclassifications could have been caused by two probable reasons. One reason is that a transition epoch from NE to AE or AE to NE is difficult to classify. For example, Figure 5 shows a transition epoch from NE to AE, whereas Figure 6 shows a transition epoch from AE to NE. A skilled physician would be able to classify these epochs based on the contextual information. However, the proposed model does not use the contextual information to score OSA, making it unable to distinguish the transition epochs. The other reason may be that the proposed model finds it difficult to score the artifact epochs. The ECG signals can be polluted by unwanted noise signals, including body movement.  Figure 7 shows a polluted ECG epoch. Because the artifact epochs are few and varied, the proposed model was unable to learn the distributions of all the artifact epochs. Therefore, it is difficulty for the proposed model to detect the OSA of artifact epochs. In this case, the usage of handcrafted features seems to be considerably robust.

### 4.4. Benefits of Long Short-Term Memory

The major advantage associated with the usage of LSTM is that it can be trained to learn long-term dependencies, including the transition rules that are used by the physicians to identify the next possible OSA event(s) from a sequence of ECG epochs. To validate the usefulness of LSTM, we removed the LSTM layer from the model (Figure 2) and then reimplemented the experiment. This test was named CNN_1.  Table 5 shows the comparison results, where we can see that the proposed model (CNN + LSTM) results in a gain of 1.3% over the TAC of CNN_1. In addition, KP increased by 0.03 when LSTM was added, verifying our assumption.

**Figure 8:**
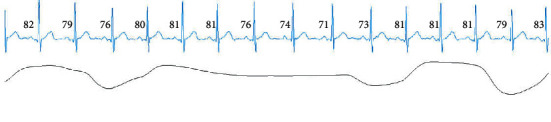
A normal ECG epoch.

Figure 8 shows an example of the NE ECG signal. When the proposed method (CNN + LSTM) is used, the epoch is classified as an NE. However, when CNN_1 is used, this epoch is scored as an OSA event. The reason for OSA misclassification is that the heart rate is slow at the center of this epoch. According to a previously conducted study [11], the heart rate decelerates when OSA occurs. Therefore, CNN_1 learned this feature. However, from Figure 8, we can observe that the heart rate changes very little. At the same time, the heart rates of previous epochs are similar to those of this epoch. However, because the LSTM learns long-term dependencies, the CNN + LSTM method accurately detects the epoch, which is the benefit associated with the usage of LSTM.

### 4.5. OSA Detection

**Figure 9:**
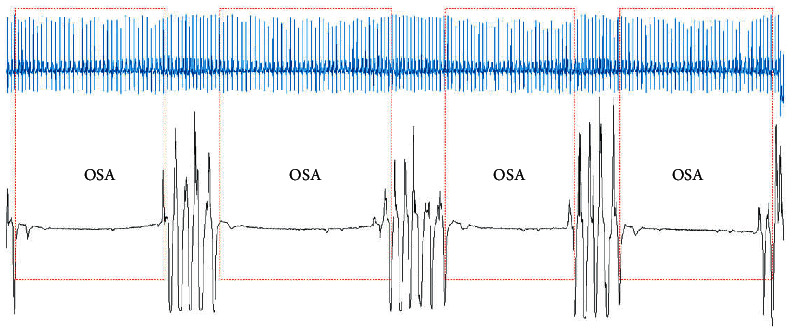
The start and end positions of multiple OSA events.

As mentioned previously, long OSA is dangerous because it can lead to sudden death. To identify long OSA, the window overlapping method can be used to detect the start and end positions of an OSA event. In this way, long OSA can be detected Figure 9 shows that the proposed model can detect complete OSA events from the ECG signals. From the nasal airflow signal, we can observe that the OSA events detected by our model have been accurately identified.

### 4.6. Comparison of the Proposed Method with Existing Studies

The comparison of various methods of automatic OSA detection is difficult because different datasets, feature sets, and classifiers are used in different studies. For ensuring a fair comparison with existing studies, Table 6 shows the classification performances of different methods based on single-lead ECG signals. From Table 6, we can observe that the proposed model achieved better performance when compared with those achieved in the previous studies. More importantly, our method can be used in conjunction with wearable medical devices, which is very important for home OSA monitoring.

## 5. Conclusions

In this study, we developed an automated OSA event detection method using a CNN, where the feature extraction and selection processes were not required. The proposed method detected the start and end positions of the OSA events based on the overlapping epochs in the ECG signal dataset. Our method automatically extracted the time-invariant features from raw ECG signals without utilizing any handcrafted features. The proposed approach is robust and completely automated, and the method can be easily adapted to other physiological signal analyses and prediction problems. The TAC and KP of the proposed model applied to the single-channel ECG reached 96.1% and 0.92, respectively. The experimental results showed that the proposed method could accurately score the OSA events and that it achieved comparable performance with other state-of-the-art studies. More importantly, our method can prevent sudden death from OSA, which is important for the patients who are severely affected by OSA.

There are some limitations associated with our CNN method. First, the proposed model can only detect OSA and normal events but not hypopnea events. Although hypopnea is not as serious as OSA, it is still prevalent in sleep-disordered breathing patients. Second, it is difficult to score transition epochs using our method. In the future, we will improve the discrimination ability of our method for AEs and NEs. In addition, the automated anomaly detection of ECG based on the CNN, which is important to rapidly assess the quality of the ECG data, will be studied.

## Figures and Tables

**Table 1:** The number of normal epochs (NE) and abnormal epochs (AE).

Training set		Test set	
NE	AE	NE	AE
210680	130050	213830	13102

**Table 2:** The parameters and TACs of the different models.

Name	*N*	Layer	Units	Size	Stride	TAC (%)
Model_1	3	Cn_1	24	125 × 1	1 × 1	94.832
		Cn_2	24	15 × 1	1 × 1	
		Cn_3	24	5 × 1	1 × 1	
Model_2	4	Cn_1	24	125 × 1	1 × 1	94.835
		Cn_2	20	100 × 1	1 × 1	
		Cn_3	24	15 × 1	1 × 1	
		Cn_4	24	5 × 1	1 × 1	
Model_3	4	Cn_1	24	125 × 1	1 × 1	93.92
		Cn_2	20	50 × 1	1 × 1	
		Cn_3	20	15 × 1	1 × 1	
		Cn_4	20	5 × 1	1 × 1	
Model_4	3	Cn_1	24	100 × 1	1 × 1	94.78
		Cn_2	24	15 × 1	1 × 1	
		Cn_3	24	5 × 1	1 × 1	
Model_5	2	Cn_1	30	125 × 1	1 × 1	90.4
		Cn_2	30	15 × 1	1 × 1	

**Table 3:** The parameters of the proposed model.

Layer	Layer type	Units	Unit type	Size	Stride	Output size
Input						1000 × 1
BN						1000 × 1
Cn_1	Convolutional	24	ReLU	125 × 1	1 × 1	876 × 24
Cn_2	Convolutional	24	ReLU	15 × 1	1 × 1	986 × 24
Cn_3	Convolutional	24	ReLU	5 × 1	1 × 1	996 × 24
Mp_1	Max pooling	24		2 × 1	1 × 1	438 × 24
Mp_2	Max pooling	24		2 × 1	1 × 1	493 × 24
Mp_3	Max pooling	24		2 × 1	1 × 1	498 × 24
Concatenate		24				1429 × 24
Mp_4	Max pooling	24		3 × 1	1 × 1	476 × 24
Add	Add	24				1000 × 24
Dense	Fully connected	48	LeakyReLU			1000 × 48
Dropout	Dropout					1000 × 48
Gp	Global pooling					48 × 1
LSTM	LSTM					64 × 1
Dense	Fully connected	2	Softmax			2

**Table 4:** The performances of the proposed model for automated detection of OSA.

	NE	AE	KP	SE (%)	SP (%)	TAC (%)	PPV (%)	NPV (%)
NE	202460	4940	0.92	96.1	96.2	96.1	97.6	93.8
AE	8220	125110						

**Table 5:** Comparison of classification performances.

Model	KP	TAC (%)
CNN_1	0.89	94.8
CNN + LSTM	0.92	96.1

**Table 6:** Comparison of performances of different methods.

Input	Author	Method	TAC (%)	SE (%)	SP (%)
ECG	Jafari [43]	Handcrafted features, SVM	94.8	94.1	95.4
	Chen et al. [44]	Handcrafted features, SVM	82.1	83.2	80.2
	Urtnasan et al. [25]	CNN	96	96	96
	Banluesombatkul et al. [34]	CNN	79.45	77.6	80.1
	Zarei and Asl [12]	Handcrafted features, SVM	94.63	94.43	94.77
	Tripathy [45]	Handcrafted features, kernel extreme learning machine	76.37	78.02	74.64
	Hassan and Haque [46]	Handcrafted features, RUSboot	88.88	87.58	91.49
	Hassan [47]	Handcrafted features, AdaBoost	87.33	81.99	90.72
	Our method	CNN	96.1	96.1	96.2

## Data Availability

The Apnea-ECG dataset, downloaded from https://www.physionet.org/content/apnea-ecg/1.0.0, was used to evaluate our proposed approach.
